# Reduction of breathing irregularity-related motion artifacts in low-pitch spiral 4D CT by optimized projection binning

**DOI:** 10.1186/s13014-017-0835-7

**Published:** 2017-06-19

**Authors:** René Werner, Christian Hofmann, Eike Mücke, Tobias Gauer

**Affiliations:** 1 University Medical Center Hamburg-Eppendorf, Martinistr. 52, Hamburg, 20246 Germany; 2 Siemens Healthcare GmbH, Siemensstr. 1, Forchheim, 91301 Germany

**Keywords:** 4D CT, Motion artifacts, Breathing irregularity, Artifact reduction

## Abstract

**Background:**

Respiration-correlated CT (4D CT) is the basis of radiotherapy treatment planning of thoracic and abdominal tumors. Current clinical 4D CT images suffer, however, from artifacts due to unfulfilled assumptions concerning breathing pattern regularity. We propose and evaluate modifications to existing low-pitch spiral 4D CT reconstruction protocols to counteract respective artifacts.

**Methods:**

The proposed advanced reconstruction (AR) approach consists of two steps that build on each other: (1) statistical analysis of the breathing signal recorded during CT data acquisition and extraction of a patient-specific reference breathing cycle for projection binning; (2) incorporation of an artifact measure into the reconstruction. 4D CT data of 30 patients were reconstructed by standard phase- and local amplitude-based reconstruction (PB, LAB) and compared with images obtained by AR. The number of artifacts was evaluated and artifact statistics correlated to breathing curve characteristics.

**Results:**

AR reduced the number of 4D CT artifacts by 31% and 27% compared to PB and LAB; the reduction was most pronounced for irregular breathing curves.

**Conclusions:**

We described a two-step optimization of low-pitch spiral 4D CT reconstruction to reduce artifacts in the presence of breathing irregularity and illustrated that the modifications to existing reconstruction solutions are effective in terms of artifact reduction.

**Electronic supplementary material:**

The online version of this article (doi:10.1186/s13014-017-0835-7) contains supplementary material, which is available to authorized users.

## Background

In radiotherapy (RT) treatment planning for thoracic and abdominal cancer patients, the term 4D CT refers to respiration-correlated computed tomography, and a 4D CT data set is understood as a series of 3D CT images of the patient geometry at different breathing states. Since the seminal works in this field [1–3], 4D CT has rapidly found its way into clinical practice [4] and is currently estimated to be routinely applied in approximately 70% of the RT centers in the US [5]. 4D CT information are, for instance, used to dimension the internal target volume and to perform 4D dose calculation in the context of 4D RT quality assurance [6–9]. Very recently, 4D CT imaging has even been reported to be applied for CT ventilation image-guided RT treatment, exploiting a registration-based local lung volume change analysis in the 4D CT images and feeding this information back into treatment plan optimization [5].

All 4D CT use cases have in common that their reliability depends on 4D CT image quality and the absence of motion artifacts [6, 7, 10, 11]. However, in agreement with earlier publications [12], a retrospective analysis of our in-house 4D CT database of more than 50 patients treated between 2012 and 2014 revealed a fraction of 75% of the images being subject to artifacts [13], and consequently still motivates improving existing 4D CT reconstruction.

The principle of current commercial 4D CT protocols is to acquire a respiratory signal recorded using, for instance, abdominal belts or camera-based tracking of markers positioned on the patient’s chest wall. The recorded breathing signal is then correlated to the simultaneously acquired projection or image data. These ‘tagged’ CT data are finally sorted and/or reconstructed according to the assigned breathing state information, resulting in the desired series of 4D CT images at different breathing states. Although acquisition and reconstruction details vary between the CT vendors, they all face the problem that standard RT CT systems are not capable of scanning the entire anatomical region of interest within a single gantry rotation. As a consequence, data acquisition spans multiple breathing cycles and the acquired CT data has to be appropriately pieced together. Thus, assumptions of 4D CT protocols are

1. regular breathing patterns during scanning,
2. a constant relationship between breathing signal and internal anatomical motion, and
3. the data sufficiency condition (DSC, i. e. existence of sufficient CT data to reconstruct images at all desired breathing states [14]).

Violations of above assumptions lead to often seen motion artifacts like incomplete and double structures [15].

Turning partly away from common 4D CT data acquisition, Thomas et al. described that it is in principle possible to generate artifact-free 4D CT images by repeat fast spiral scanning and subsequent registration- and model-based reconstruction [16]. Although we consider the solution promising, it will still take some time until related protocols are ready for clinical use [11]. Thus, our question was whether we have, *at the moment* and in clinical practice, to work with the artifact-affected image data – or whether there are ways to mitigate artifacts using *current* clinical 4D CT protocols and modifications thereof.

Our study focusses on standard external breathing signal-driven low-pitch spiral 4D CT [1], i.e. the CT table feed per gantry rotation has to be sufficiently low to ensure existence of an appropriate amount of projection data to allow for reconstruction of CT images at the desired breathing states. We, however, aimed at implementation and evaluation of generally applicable and quickly to reimplement modifications to existing 4D CT scanning and reconstruction protocols (including ciné protocols) that were assumed to have a relevant impact in terms of an artifact reduction. In detail, we propose a two-step modification to counteract artifacts due to violations of A1 and A2. It consists of an optimized breathing state definition and projection binning to combine advantages of well-known amplitude- and phase-based sorting algorithms (first step), together with an image-domain artifactness measure that is to be minimized during the 4D CT image reconstruction (second step). The first step extends classical work on optimized breathing state definition for 4D CT reconstruction [17, 18] by incorporating a statistical analysis of the patient-specific breathing record acquired during CT scanning and a subsequent patient-specific reference curve-based breathing state definition. The second step is intended to account for remaining uncertainties due to, e.g., changes in the relationship between the external breathing signal and the internal anatomical motion (violations of A2). Starting with an initial assignment of the aforementioned breathing states to the acquired CT projection data, the idea is to slightly vary the actual projection data used for reconstruction of the transversal CT slices for the individual breathing states and to search for a combination of slices that minimizes the artifactness measure. The concept of varying projection data intervals used for reconstruction to reduce motion artifacts has, for instance, been successfully utilized by O’Brien et al. for improved 4D Cone Beam (CB) CT reconstruction [19]. Their approach is, however, limited to the specific challenges of 4D CBCT and especially the presence of streaking artifacts due to projection undersampling; the respective objective function to be minimized can therefore not directly be transferred to standard 4D CT reconstruction and artifacts. Similar thoughts and promising results can, nevertheless, also be found in the context of ciné 4D CT image sorting [20]; the respective work forms the basis of our implementation. Thus, with the design of the two steps of our approach for reduction of breathing irregularity-related 4D CT motion artifacts being motivated by recent developments in the context of 4D CT imaging, we present a novel combination thereof and describe their adaptation to low-pitch spiral 4D CT. The potential of our optimized projection binning algorithm is evaluated by means of 30 clinical 4D CT data sets; a comparison to common phase- and amplitude-based sorting and 4D CT reconstruction illustrates the proposed 4D CT reconstruction modifications to significantly reduce motion artifacts.

## Methods

### Advanced reconstruction (AR) algorithm

As mentioned before, the advanced reconstruction consists of two steps, which build on each other and are detailed below.

#### AR step 1: Reference curve-based projection binning

**Fig. 1:**
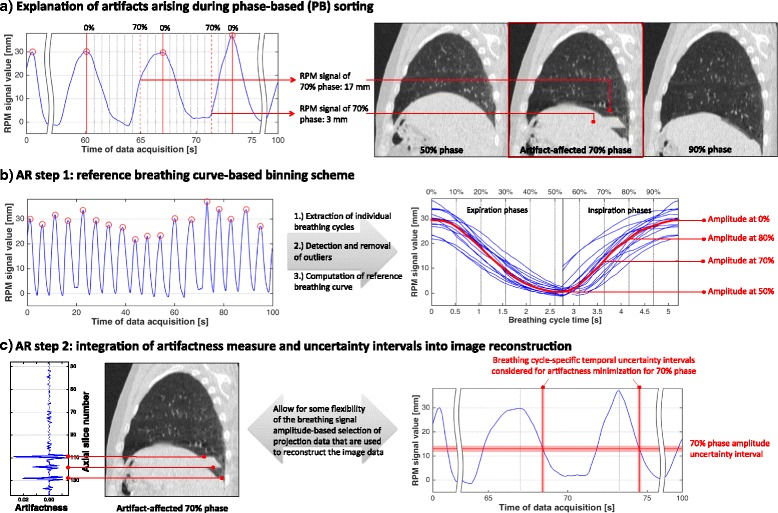
Illustration of addressed problem and sketch of proposed solution. **a** Typical 4D CT artifacts stem from inappropriate breathing state definition and/or assignment to projection data (in low-pitch spiral 4D CT) or reconstructed image segments (ciné 4D CT). **b** To overcome shortcomings of classical phase- or amplitude-based sorting approaches, we extracted a patient-specific reference breathing curve that was used for phase- and breathing signal amplitude-assignment to the acquired projection data. **c** In addition, an artifactness measure was introduced into and to be minimized during image reconstruction. To retain the range of breathing dynamics represented in the acquired data, uncertainty intervals were defined to restrict the minimization search space

Standard clinically available projection binning approaches are phase-based (PB) and amplitude-based (AB) sorting; using a Siemens CT, the latter is implemented as local amplitude-based (LAB) sorting [21]. PB sorting means that each breathing cycle of the acquired respiratory signal is split into *n*_*ph*_ points or bins (*n*_*ph*_: number of breathing states to reconstruct 3D CT images at; typical: *n*_*ph*_≈10) that are equidistantly distributed in time. In the case of inter-cycle waveform variability, this leads to the situation that the breathing states that correspond *per definition* do not agree regarding the respective *actual* physiological state. If these inappropriate states are used to tag the projection data for image reconstruction, the differences in the actual breathing states manifest as artifacts, as illustrated in Fig. 1 a. In LAB sorting, each breathing cycle is equidistantly sampled with respect to the breathing signal amplitude. Amplitude sorting reconstruction is less prone to motion artifacts [17, 18]; variations in the depth of breathing, however, still affect AB sorting. In addition, equidistant temporal sampling – only provided by PB sorting – is often desired for RT treatment planning (e. g. to compute mid-ventilation or -position images [22]).

Aiming to combine the advantages of the two sorting approaches, we implemented a reference curve-based binning. First, the acquired breathing curve was analyzed with regard to the end-inspiration signal amplitudes; CT projection data corresponding to breathing signal amplitudes higher than the mean end-inspiration signal amplitude were discarded and not used for reconstruction purposes. Similar to respective work in the context of global AB sorting [18], this counteracts artifacts due to pronounced variations in the depth of breathing. To finally derive a patient-specific representative breathing cycle, the individual breathing cycles were temporally aligned at the end-inspiration peak, Fig. 1 b. After alignment and excluding ‘outlier’ breathing cycles (peak-to-peak amplitude larger then twice the mean peak-to-peak amplitude), a statistically representative breathing cycle was computed by averaging temporally corresponding signal amplitudes of the individual breathing cycles.

The resulting representative cycle was sampled equidistantly in time and the signal amplitudes of the sampling points were used to label the CT projection data for image reconstruction of the desired 3D CT volumes. Thus, effectively, an amplitude sorting variant is implemented, with the selected amplitude bins representing a temporally equidistant sampling.

#### AR step 2: Integrating ‘artifactness’ into image reconstruction

While the reference cycle-based binning approach mitigates effects due to violations of the introductory assumption A1, a potential variability of the correlation between the external breathing signal and internal motion remains challenging. Addressing violations of A2 in the context of ciné 4D CT imaging, Castillo et al. suggested integrating a dissimilarity measure between axial image segments acquired at spatially adjacent couch positions and the minimization of the measure into 4D CT formation [20]. Their presented workflow is, however, dependent on principles of ciné 4D CT imaging, i. e. a consecutive acquisition and reconstruction of a series of image segments for fixed couch positions, breathing signal-based assignment of respiratory states to already reconstructed image segments, and the assembly of the image segments of a similar breathing state but different couch positions.

However, the core idea of Castillo et al. appears to be transferable to low-pitch spiral 4D CT. In a nutshell, they allowed for more flexibility during the assembly of image segments of different couch position: While the standard approach during image segment assembly is to select *the* single image segment for each couch position that, in terms of the assigned breathing state, is closest to the desired one, they considered three image segments of similar breathing state labels per couch position. Finally, taking into account all image segment combinations, segments were stacked together that – across the entire 3D CT image at the desired breathing state – minimized the mentioned inter-segment image dissimilarity.

Given its promising ciné 4D CT performance (reduction of the dissimilarity measure by 24% compared to standard PB reconstruction), we transferred and adapted their approach to low-pitch spiral 4D CT and combined it with the described reference curve binning approach. In contrast to ciné 4D CT, low-pitch spiral 4D CT comprises breathing state assignment to the acquired CT projection data (rather than already reconstructed image segments); the continuous patient transport during spiral raw data acquisition and the associated need for synthesized planar projection data lead to additional challenges. At this, aiming at adapting the presented workflow as directly as possible and with as little interference with the actual and vendor-specific CT image reconstruction process as necessary, we decided to introduce time stamp *uncertainty intervals* with respect to the projection data breathing state labels obtained by the reference curve-based binning.

In detail, let the breathing state labels that sample the patient-specific reference cycle range between 0% and 100%, with 0% (= 100%) denoting end-inspiration. Thus, labels for a 10-state reconstruction are 0%, 10%, …, 90%, which correspond to patient-specific inspiration and expiration breathing signal amplitude values; see Fig. 1 b. To account for violations of A2 and related uncertainties of the breathing state tags assigned to the projection data, we introduced uncertainty intervals of ±5% with respect to the breathing state-specific signal amplitude values. These intervals and respective couch-position specific time periods, as shown in Fig. 1 c for a 70% state, were then equidistantly sampled and corresponding axial images reconstructed. This means that for each couch position and breathing phase a set of candidate image slices was reconstructed (here: between five and seven slices), with the breathing states represented by the candidate slices being slightly varied.

Now, similar to Castillo et al., a 3D image volume at a specific breathing state was assembled by combining slices of the candidate slice sets of the different couch positions in such a way that a dissimilarity or ‘artifactness’ measure between adjacent image slices was minimized. The exploited artifactness measure is illustrated in Fig. 1 c and was adopted from a work on automated artifact detection in ciné 4D CT data [23]. Formally speaking, let $I:\Omega \subset \mathbb {Z}^{3}\rightarrow \mathbb {Z}$ denote a 3D CT data set and *I*(*x,y*,*z*) the CT value at voxel position (*x,y*,*z*). Further, $I_{\hat {z}}=I|_{\Omega _{\hat {z}}}$ represents the restriction of the 3D image *I* to $\Omega _{\hat {z}}=\{\left (x,y,z\right)\in \Omega \ |\ z={\hat {z} }\}$, i. e. $I_{\hat {z}}$ defines the axial slice of *I* at couch position $\hat {z}$. Then, the artifactness of $I_{\hat {z}}$ was computed as

$$ {}C\left[I_{\hat{z}}\right] \,=\, \frac{1}{2}\! \left(\!C^{\rm{CC}}\!\left[I_{\hat{z}-1},I_{\hat{z}}\right]\,+\,C^{\rm{CC}}\!\left[I_{\hat{z}+1},I_{\hat{z}+2}\right]\!\right)-C^{\rm{CC}}\left[I_{\hat{z}},I_{\hat{z}+1}\right] $$

with

$$ {} C^{\rm{CC}}\left[I_{\hat{z}},I_{\hat{z}'}\right]= \frac{1}{|\Omega_{\hat{z}}|}\sum\limits_{\left(x,y\right)\in\Omega_{\hat{z}}}{\frac{\left(I_{\hat{z}}\left(x,y\right)-\overline{I_{\hat{z}}}\right)\left(I_{\hat{z}'}\left(x,y\right)-\overline{I_{\hat{z}'}}\right)}{\sigma\left[I_{\hat{z}}\right]\sigma\left[I_{\hat{z}'}\right]}} $$

measuring the normalized cross-correlation (CC) of two slices $I_{\hat {z}}$ and $I_{\hat {z}'}$ of *I*. Here, $\overline {I_{\hat {z}}}$ is the mean CT value of slice $I_{\hat {z}}$ and $\sigma \left [I_{\hat {z}}\right ]$ the corresponding standard deviation. Thus, *C* ^*CC*^ ranges between -1 and +1, with +1 indicating perfect positive correlation between the HU values of the two compared slices; consequently, the closer the value is to +1, the higher is the similarity between the slices. However, direct application of *C* ^*CC*^ does not allow for reliable identification of artifacts because of potential co-localized differences between the anatomical structures represented in the two slices that also influence *C* ^*CC*^. To address this issue and to be able to assign an artifactness to each single image slice, the cross correlation of the considered slice and its direct successor slice is evaluated with respect to corresponding values in a small neighborhood of the considered slice. This is expressed by the definition of $C\left [I_{\hat {z}}\right ]$ and finally means that high positive peaks of *C* indicate that the respective slices are very likely to be subject to image artifacts. Further details on the artifactness measure can be found in the underlying publication [23].

### Study design: Image data and experiments

Our study was based on 4D CT data sets of 30 patients with lung and liver lesions. All images were acquired with a Siemens Definition AS+ system (Siemens Healthcare, Germany) in spiral mode and with a retrospective respiratory protocol. Imaging parameters were the same for all patients: 0.5 s gantry rotation time, 0.09 pitch factor, breathing signal acquisition using the Real-Time Position Management system (Varian Medical Systems, USA). The acquired projection data were reconstructed into 10-state 4D CT images applying the PB and the LAB options clinically available for our scanner. The LAB option is routinely applied in our clinic; respective images of the considered patients were used for treatment planning. In addition, 4D CT images were computed using the advanced reconstruction (AR) approach proposed in this paper.

As a first analysis part, the 4D CT data sets were divided into two groups. Group 0 were the 4D CT data sets with – according to a rapid visual assessment of the LAB data – only small artifacts; group 1 contained the remaining artifact-affected data sets. In addition, the breathing signals acquired during 4D CT data acquisition were analyzed and parameters extracted that were assumed to characterize the breathing curves and their irregularity: mean and standard deviation of the peak-to-peak breathing cycle amplitude; mean, minimum, maximum and standard deviation of the breathing cycle lengths; standard deviation of the cycle-specific minimum and maximum signal values; and the slope of a fitted linear signal baseline drift. Nominal logistic regression was applied to analyze the relation between these entities and group membership (membership as binary dependent variable) and to identify parameters that significantly contribute to the explanation of group membership.

For quantitative comparison of the performance of the reconstruction approaches, all reconstructed images were then subject to a thorough visual inspection: The total number of artifacts in the images was counted in coronal and sagittal views of the images. To further evaluate the importance of the individual AR steps, we then selected the ten patients with largest difference in the number of artifacts after LAB and AR; for these patients, additional data sets were reconstructed using only AR step 1 (i.e. no minimization of the artifactness measure has been applied). To avoid evaluation inaccuracies due to inter-observer variability, all data sets were evaluated by a single experienced observer; however, differences between two raters were also evaluated by means of the artifact numbers in the AR images. Statistical significance of differences between two reconstruction approaches was tested by paired t-tests; multiple testing was accounted for by Bonferroni correction.

After evaluation of the number of motion artifacts, the respective differences between the reconstruction methods were correlated to the entities that, according to the aforementioned logistic regression analysis, were significantly related to group 1 membership (partial correlation, accounting for the total number of artifacts per patient as potentially confounding covariate). The goal was to verify the hypothesis that AR predominantly counteracts formation of artifacts in the presence of breathing irregularity and to identify respective breathing signal parameters.

The last part of our study aimed at identification of fields of future work: For the ten patients with the largest number of remaining motion artifacts after AR, the residual artifacts were analyzed and categorized.

## Results

The results section is divided into two subsection: The comparison results of the performance of the reconstruction approaches are subsequently described. Afterwards and as outlined in the study design, detailed information on the correlation of artifact reduction and breathing characteristics are presented.

### Quantitative evaluation of AR-related artifact reduction

Table 1 summarizes the number of artifacts in the image data reconstructed by the different approaches and respective differences. While PB and LAB images contained, averaged over all breathing states and patients, a mean number of 4.8 and 4.6 artifacts, the AR images had on average 3.3 artifacts; this corresponds to a significant reduction of 31.1% and 27.4% (*p* <0.001). The detailed analysis of the ten patient data sets with the largest difference in the number of artifacts after LAB and AR further revealed that AR step 1 accounts for approximately two-thirds of the AR-related artifact reduction.

**Table 1:** Results of the evaluation and comparison of the number of artifacts in the reconstructed images (EI = end inspiration; EE = end-expiration)

		Breathing state-specific evaluation (max. inspiration to max. inspiration)									
		EI	← Expiration states →				EE	←Inspiration states →			
Reconstruction	Mean	0%	10%	20%	30%	40%	50%	60%	70%	80%	90%
Number of motion artifacts (mean ± standard deviation over patients)											
Phase-based (PB)	4.8±2.2	5.8±2.9	5.2±2.8	5.2±2.4	4.1±2.5	3.7±2.6	3.7±2.3	4.3±2.6	5.4±2.4	5.6±2.4	5.4±2.9
Amplitude-based (LAB)	4.6±2.1	5.8±2.6	5.2±2.4	4.9±2.6	4.6±2.6	3.7±2.4	3.6±2.1	4.0±2.5	4.5±2.3	4.5±2.3	5.0±2.8
Advanced recon. (AR)	3.3±1.7	3.6±2.5	3.3±2.1	3.6±2.1	3.2±1.9	3.0±1.9	2.6±1.9	3.4±2.0	3.3±2.0	3.3±1.9	4.1±2.3
Average reduction of artifacts by first mentioned approach compared to second approach											
LAB vs. PB	5.1*%*	−0.6*%*	0.0*%*	5.8*%*	−12.2*%*	0.0*%*	2.7*%*	6.2*%*	16.7*%* ^∗^	20.2*%* ^∗^	7.7*%*
AR vs. LAB	27.4*%* ^∗^	38.5*%* ^∗^	36.3*%* ^∗^	27.2*%* ^∗^	29.7*%* ^∗^	20.5*%* ^∗^	27.1*%* ^∗^	16.5*%*	27.4*%* ^∗^	26.9*%* ^∗^	18.1*%*
AR vs. PB	31.1*%* ^∗^	38.2*%* ^∗^	36.3*%* ^∗^	31.4*%* ^∗^	21.1*%* ^∗^	20.5*%*	29.1*%* ^∗^	21.7*%* ^∗^	39.5*%* ^∗^	41.7*%* ^∗^	24.4*%* ^∗^

^∗^Differences are significant on 5% significance level

In contrast to the comparison of AR to PB and LAB reconstruction, the 5.1% difference between LAB and PB was not significant (*p* =0.13), and neither were differences of the number of artifacts counted in the AR images by the two observers significant (inter-rater differences = 4.9%; *p* =0.44).

**Fig. 2:**
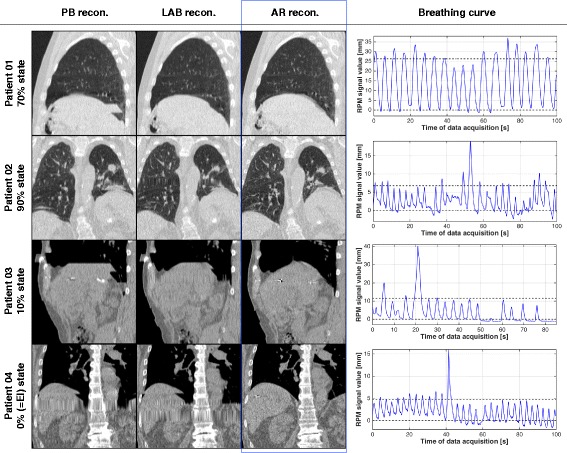
Comparison of the considered reconstruction approaches. Artifacts were reduced by the advanced reconstruction approach AR for a wide range of breathing irregularity (compare breathing curves on the right hand side). The effect is, however, most pronounced in the case of relatively irregular breathing patterns and usually most visible in images at breathing states close to the end-inspiration state

As the number of artifacts does not necessarily reflect the visual impression of a potential reduction of residual artifacts, example coronal and sagittal CT slices are shown in Fig. 2. Corresponding movies that represent all reconstructed breathing states are provided as Additional files 1, 2, 3 and 4 to this paper.

### Correlation of artifact reduction and breathing characteristics

The breathing state-specific information in Table 1 shows that the number of artifacts (especially for PB and LAB) as well as the artifact reduction by AR are largest close to end-inspiration. It is further known that end-inspiration is not as reproducible as the end-expiration state [24]; thus, the numbers in the table can already be interpreted as an evidence that AR indeed counteracts artifacts arising due to breathing irregularity.

In line, the logistic regression analysis reveals that group 1 membership (presence of many and/or very pronounced artifacts; 14/30 data sets) was significantly related to – and only to – larger standard deviations of the breathing amplitude and the end-inspiration peak values of the acquired breathing signals, in conjunction with a prolonged mean breathing period (*p* <0.05 for the model comparison with and without these entities; McFadden’s pseudo-R^2^ of 0.52 for the entire model further indicates an overall good fit of group membership).

Obviously, only breathing amplitude and end-inspiration peak value standard deviations are directly related to breathing irregularity; and indeed these two factors were strongly correlated to the amount of artifact reduction obtained by AR compared to PB (Spearman correlation coefficients *ρ* of 0.68 for the breathing cycle amplitude value standard deviation and 0.65 for the standard deviation of the end-inspiration signal values; *p* =0.003 and *p* =0.006) and LAB (*ρ*=0.56 and *ρ*=0.48; *p* =0.03 and *p* =0.09). In turn, prolongation of the mean breathing period was related to the existence of artifacts but not significantly correlated to the achieved artifact reduction. This points to a methodical issue of the applied low-pitch spiral 4D CT protocol: The combination of a pitch factor of 0.09 and a gantry rotation time of 0.5 s requires in the current case breathing periods ≤5 s to fulfill the DSC. Artifacts due to DSC violations were, however, not addressed here.

### Artifacts remaining after AR artifact reduction

Remaining artifacts after AR were classified into artifacts due to the aforementioned violation of the DSC and incomplete or double structure artifacts. The latter type was further differentiated according to its location: either affecting the heart shape (i.e. artifacts that are likely due to different cardiac phases of the projection data used for reconstruction of adjacent axial slices) or artifacts located elsewhere. In total, artifacts due to the violation of the DSC accounted for 26 ± 20% and cardiac incomplete/double structure artifacts for 32 ± 14%; both types of artifacts are at least challenging to minimize using currently available commercial 4D CT scanning and reconstruction protocols. The remaining 42 ± 13% were still incomplete/double structure artifacts not located in the proximity of the heart; these can be assumed to be due to violations of A1 and A2 not resolved by the proposed AR approach.

## Discussion

Considering the still alarmingly high number of artifacts in routinely acquired 4D CT data, the current study addressed quickly to re-implement modifications of current low-pitch spiral 4D CT protocols: A patient-specific reference curve-based binning of projection data is combined with an integration of an artifactness measure to be minimized during the image reconstruction process. The results highlight the potential of the presented modifications to significantly reduce artifacts compared to standard PB and LAB reconstruction. As hypothesized, we further showed that the advanced reconstruction is especially helpful in the presence of breathing irregularity to reduce image artifacts – and so counteracts violations of common assumptions on breathing regularity (here: A1 and A2 of the introduction) underlying standard 4D CT protocols.

From a methodical perspective, the present study in parts builds on respective work in the field of ciné 4D CT [20], but represents the first attempt to transfer and adapt related concepts to low-pitch spiral 4D CT – and further contains the first comprehensive evaluation thereof.

Besides the achieved artifact reduction rates, Table 1 still indicates existence of residual artifacts after AR. As explained in the results section, this points in parts to methodical issues inherent to standard low-pitch spiral (and similarly ciné) 4D CT:

1. Violations of the DSC, i.e. artifacts due to violations of A3, result in missing projection data for reconstruction of desired phase images; this issue cannot be addressed by the proposed reconstruction approach.
2. Incomplete/double structure artifacts in the cardiac area are most likely due to different cardiac phases of adjacent slices and not (solely) caused by violation of A1 and A2. The second AR step implicitly reduces such artifacts, but as the projection data are binned according to respiratory states, such artifacts can finally not be sufficiently accounted for.

Respective residual artifacts motivate further development of alternative 4D CT concepts such as [16]. However, even alternative 4D CT concepts often rely on externally acquired breathing signals for definition of breathing states. At this, it should be noted that we observed almost half of the remaining artifacts after AR still to be common incomplete/double structure artifacts. Explanations and exact error sources (e.g. a lack of correlation between external signal and internal motion) remain to be scrutinized.

## Conclusions

The current study illustrates that motion artifacts in low-pitch spiral 4D CT that are due to irregularities of the patient motion patterns during 4D CT data acquisition can be significantly reduced by minor modifications of existing reconstruction protocols (i. e. by improving projection binning).

## Additional files

Additional file 1 Movie (animated gif; to be opened and viewed with, e. g., a standard web browser) that corresponds to the first data set shown in Fig. 2, but represents all ten reconstructed breathing phases. (GIF 13824 kb)

Additional file 2 Similar to the additional file 1, but representing the second data set shown in Fig. 2. (GIF 17101 kb)

Additional file 3 Similar to the additional file 1, but representing the third data set shown in Fig. 2. (GIF 12088 kb)

Additional file 4 Similar to the additional file 1, but representing the fourth data set shown in Fig. 2. (GIF 15360 kb)

## Abbreviations

AB: Amplitude-based [sorting]
AR: Advanced reconstruction
DSC: Data sufficiency condition
EE: End-expiration
EI: End-inspiration
LAB: Local amplitude-based [sorting]
PB: Phase-based [sorting]
RT: Radiotherapy
