# Supplementary figures and images for: Reduction of breathing irregularity-related motion artifacts in low-pitch spiral 4D CT by optimized projection binning
Source: Radiat Oncol. 2017 Jun 19;12:100. doi: 10.1186/s13014-017-0835-7 (PMC5477247; doi:10.1186/s13014-017-0835-7)

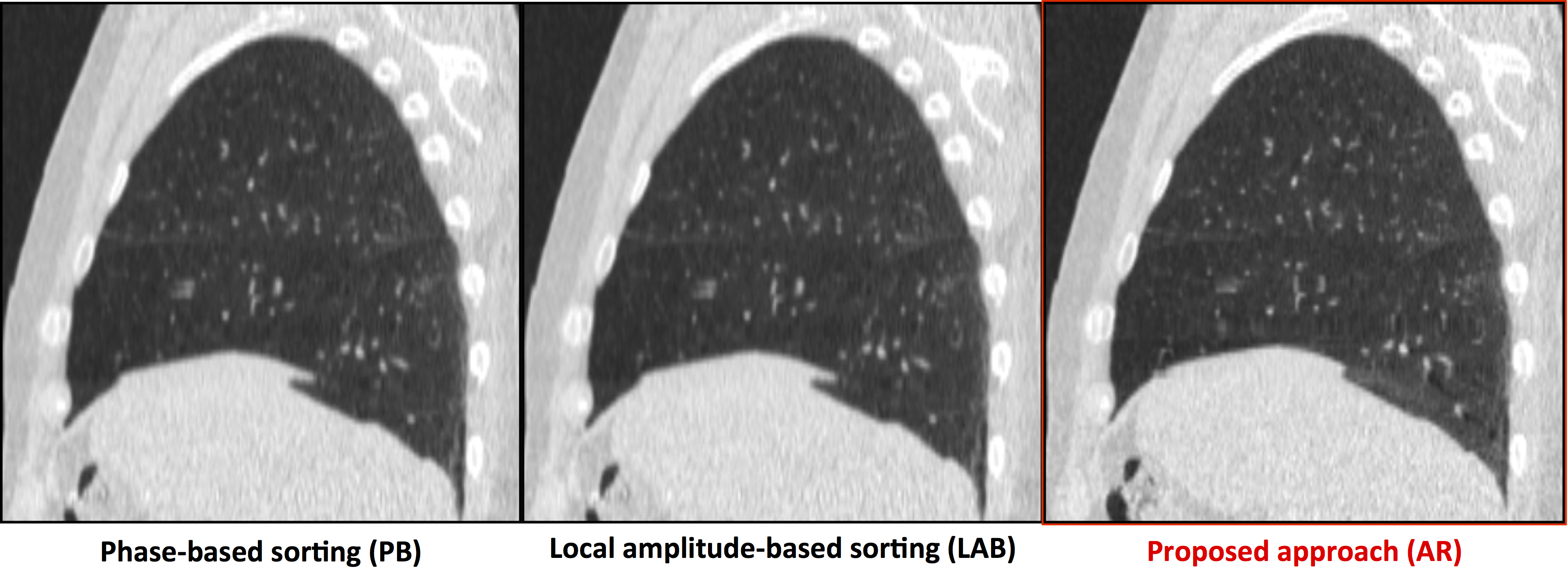

Supplement: Supplementary file 1 — Movie (animated gif; to be opened and viewed with, e. g., a standard web browser) that corresponds to the first data set shown in Fig. 2, but represents all ten reconstructed breathing phases. (GIF 13824 kb) [file 13014_2017_835_MOESM1_ESM.gif]

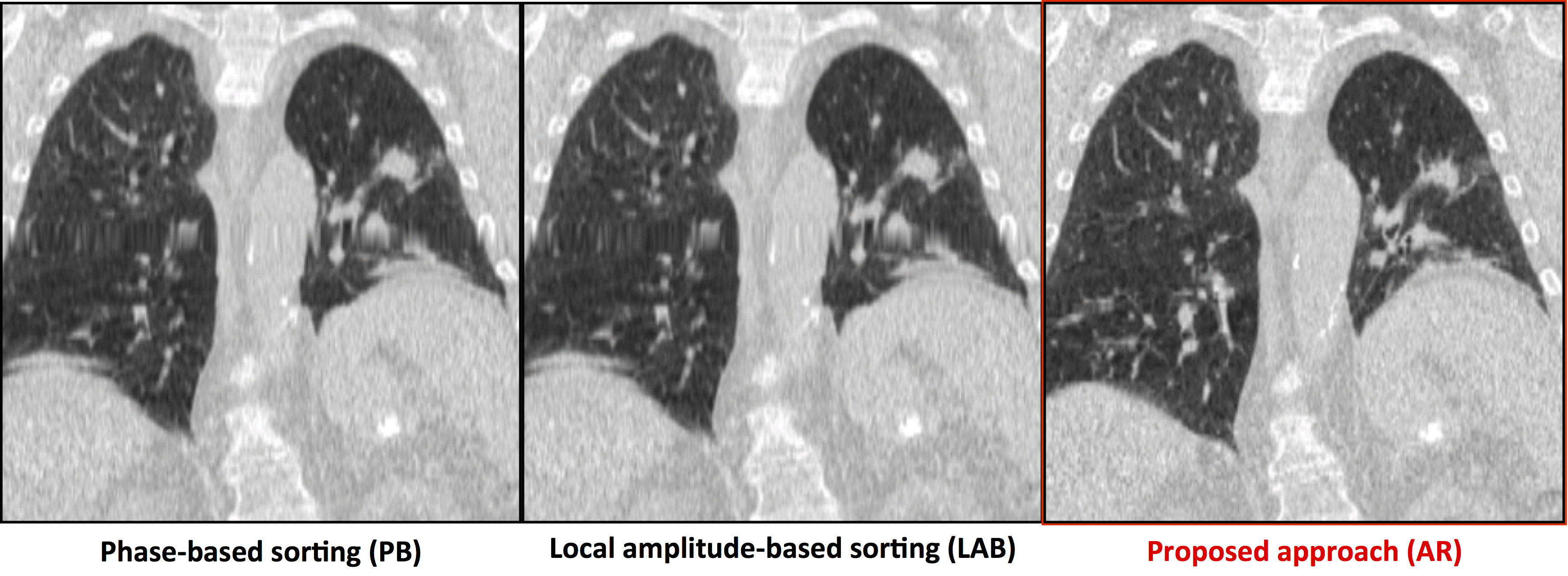

Supplement: Supplementary file 2 — Similar to the additional file 1, but representing the second data set shown in Fig. 2. (GIF 17101 kb) [file 13014_2017_835_MOESM2_ESM.gif]

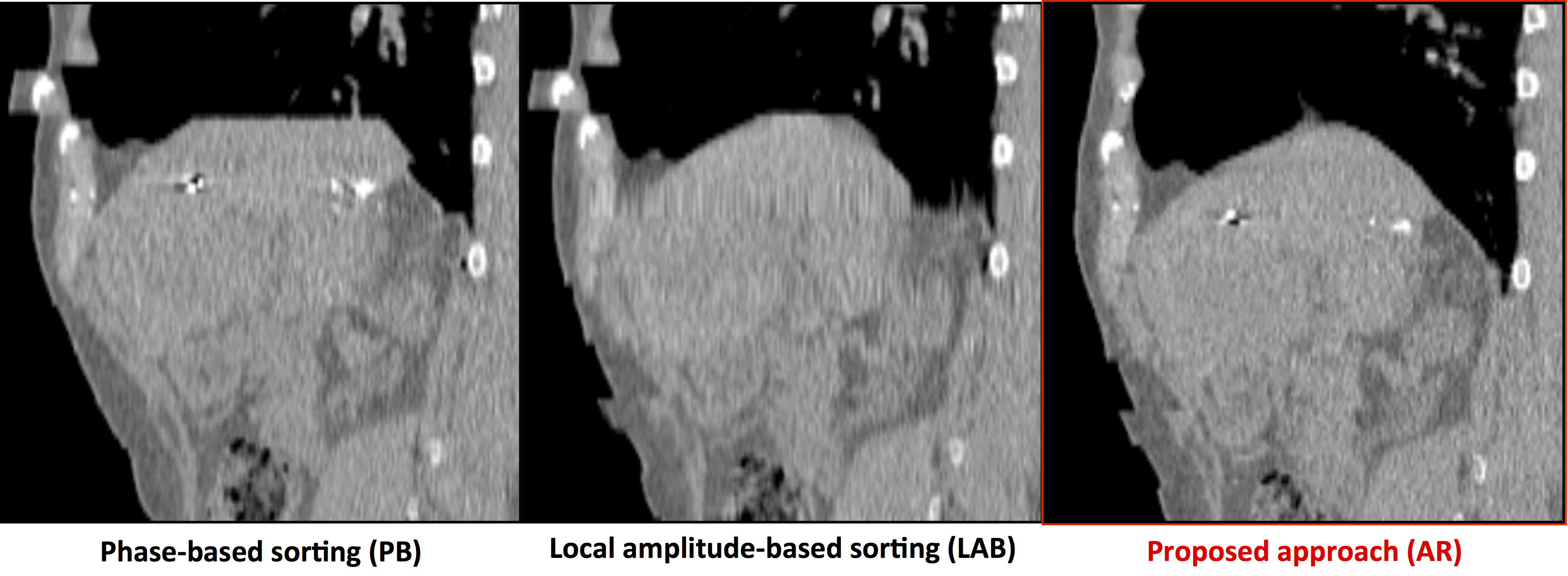

Supplement: Supplementary file 3 — Similar to the additional file 1, but representing the third data set shown in Fig. 2. (GIF 12088 kb) [file 13014_2017_835_MOESM3_ESM.gif]

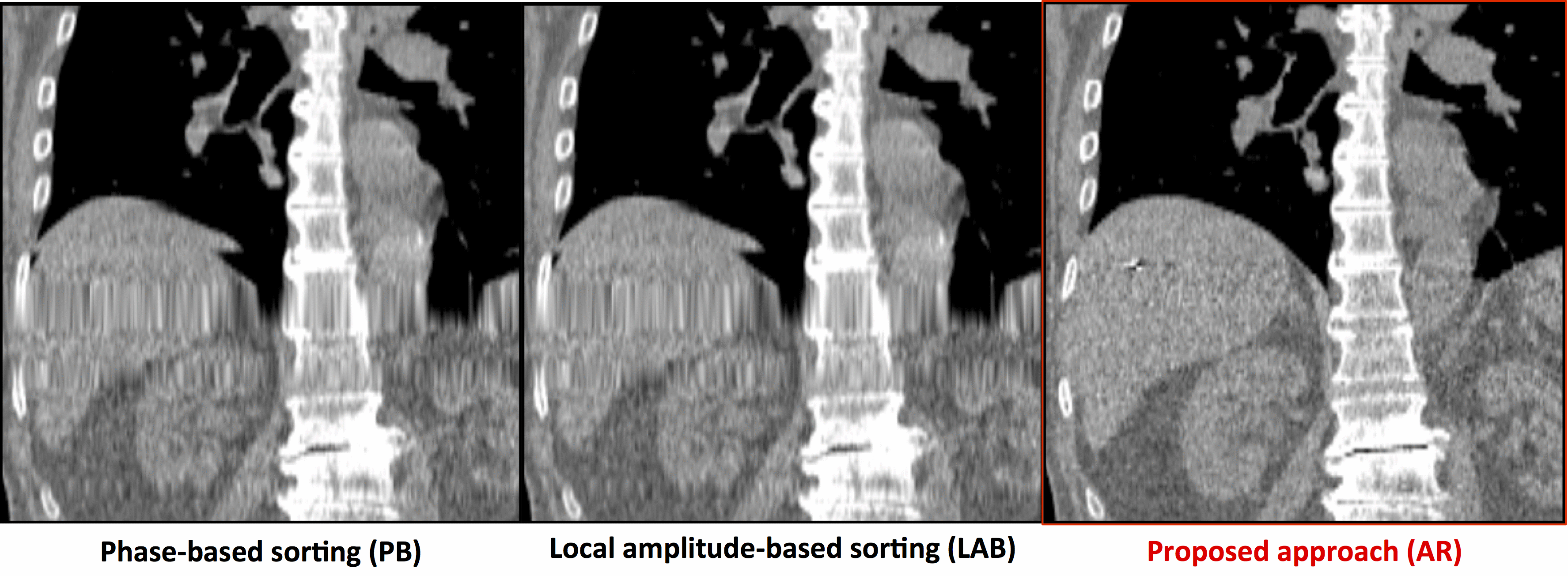

Supplement: Supplementary file 4 — Similar to the additional file 1, but representing the fourth data set shown in Fig. 2. (GIF 15360 kb) [file 13014_2017_835_MOESM4_ESM.gif]
